# The Southern Center for Maternal Health Equity (SCMHE): a multisector multifaceted community-based approach to reduce disparities in maternal morbidity and mortality in the Gulf South

**DOI:** 10.3389/fpubh.2024.1465779

**Published:** 2024-12-23

**Authors:** Melissa Goldin Evans, Maeve Wallace, Alessandra N. Bazzano, Joseph R. Biggio, Kiara Cruz, Abigail Gamble, Carmen Green, Zainab Jah, Sherri Longo, Susan Perez, Rachael N. Reed, Jeffrey G. Shaffer, Lizheng Shi, Emily Harville

**Affiliations:** ^1^Department of Social, Behavioral, and Population Sciences, Tulane University School of Public Health and Tropical Medicine, New Orleans, LA, United States; ^2^Women's Service Line, Department of Obstetrics and Gynecology, Division of Maternal Fetal Medicine, Ochsner Health System, New Orleans, LA, United States; ^3^Reproductive Health (RH) Impact, a Project of The Praxis Project, Oakland, CA, United States; ^4^Department of Preventive Medicine, John D. Bower School of Population Health, University of Mississippi Medical Center, Jackson, MS, United States; ^5^School of Population and Health Sciences, Dillard University, New Orleans, LA, United States

**Keywords:** maternal health disparities, inequities and inequalities in health, community engaged intervention, community partnered, multilevel intervention, reproductive justice (RJ)

## Abstract

**Introduction:**

The maternal mortality crisis in the United States disproportionately affects women who are Black, especially those living in the Gulf South. These disparities result from a confluence of healthcare, policy, and social factors that systematically place Black women at greater risk of maternal morbidities and mortality. This study protocol describes the Southern Center for Maternal Health Equity (SCMHE), a research center funded by the National Institutes of Health in 2023 to reduce preventable causes of maternal morbidity and mortality while improving health equity. This is a seven year program with pilot and implementation phases. SCMHE is co-led by three organizations: Reproductive Health Impact (a fiscally sponsored project of the Praxis Project), an advocacy community-based organization; Tulane University, an academic research institute; and Ochsner Health, a large regional nonprofit health system.

**Methods:**

SCMHE applies a multilevel life course approach based on the Social Ecological Model to prevent maternal morbidity and mortality with interventions at individual, interpersonal, institutional, community, and societal levels. This community-focused research center uses an intersectional lens and the Reproductive Justice framework in its aims to improve maternal health and strengthen community-based maternal health research capacity in Louisiana and Mississippi.

**Discussion:**

To advance the field of maternal health using participatory, community-centered, and radically equity-focused approaches previously underutilized and under-evaluated, the Center will lead three R01 projects to assess the implementation of existing evidence-based strategies and build the evidence base for translational research strategies.

**Ethics and dissemination:**

By leveraging our team's existing network with local, regional, and national partners while continuing to build new, unique interdisciplinary partnerships, we will build upon our distinctive interdisciplinary strengths and community connections to bring our outreach and technical assistance efforts to diverse audiences.

## Introduction

*Maternal mortality* [death from causes related to or affected by pregnancy or its management but not accidental or incidental causes (1)] is an issue of urgent concern in the United States (US) where global rankings place the US last among high-income countries (2, 3). Fundamentally a racial crisis (4), high rates are driven by the vastly disproportionate experience of death in Black families who are three to four times more likely to experience maternal mortality compared to their White counterparts (1). National trends are mirrored in Louisiana and Mississippi, where state rankings place them consistently in the top 5 for the highest rates of maternal mortality (5) and where deeply entrenched racial inequities in maternal and child health persist (6, 7).

Sadly, mortality review board findings indicate the majority of maternal deaths are preventable, (6) especially those occurring among Black women (8). Hypertensive disorders, gestational diabetes, and mental health disorders are major causes of morbidity and mortality in pregnancy, and one with a strong racial disparity as well (9, 10). Black women disproportionately experience multiple clinical and multilevel social risk factors that contribute to their elevated rates of maternal morbidity and mortality. These harms may be most pronounced within the context of the Gulf South, including Louisiana and Mississippi, where indicators of health and prosperity are worse than the rest of the country and where racial hierarchies are promoted and maintained through long-standing economic and social policies that enrich the wealthy (e.g., low taxes and few business regulations) and restrain upward mobility among Black and Brown residents (e.g., redlining, low wages, and weak safety nets) (11). Since there are higher proportions of Black residents in southern states than in the rest of the country (12), policies that differentially benefit some populations will inevitably create disparities in health and wellbeing (11, 13). For example, most states in the South elected not to expand Medicaid and thereby exacerbated coverage inequities among Black and Hispanic residents in the South (14). Additionally, inequitable access to maternal care contributes to the racial disparities in the risk of adverse maternal outcomes (15, 16).

The maternal morbidity and mortality crisis in the US is the result of a “systemic failure of current US health care, research efforts, and social policies” according to new findings from an expert panel commissioned by the National Institutes of Health (NIH) to review the evidence base (17). For example, implicit bias and racism in the healthcare system contributes to adverse maternal health outcomes, but most efforts to address such biases have been limited in scope. The expert panel developed a multilevel life course conceptual model to improve maternal health that addresses the social determinants of health and encompasses research, preventative interventions, and policy changes. This model is informed by a small but growing body of research on root causes of maternal mortality and maternal health inequities and extends beyond the biomedical model to interrogate the community, provider, systemic, structural, and policy issues of society that underlie these health outcomes (18). These causes are most easily identified by those who experience them and, indeed, may already possess the knowledge and strategies to overcome them.

**Figure 1:**
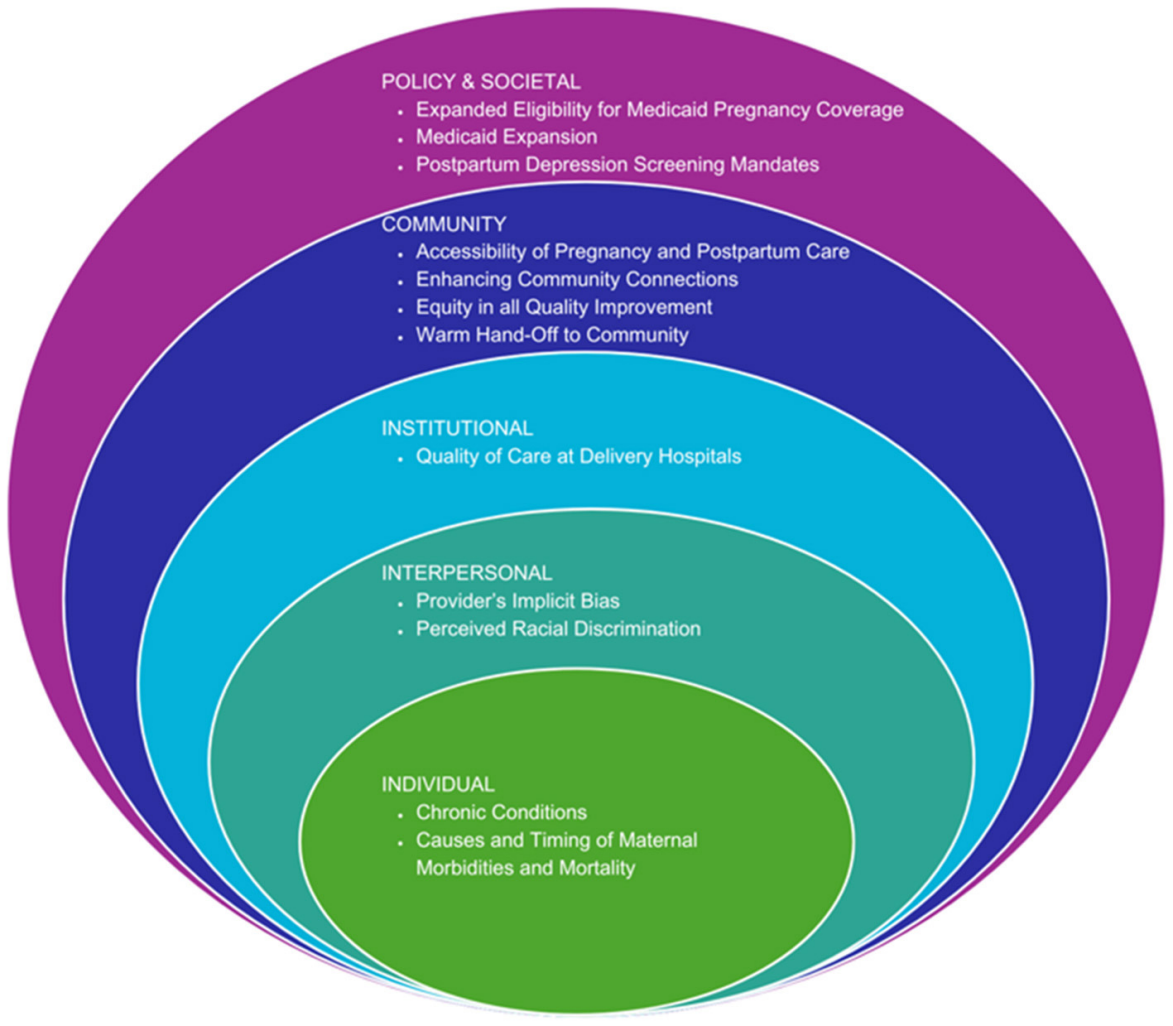
The SCMHE approach to reducing morbidity and mortality using the social ecological model.

The Southern Center for Maternal Health Equity (SCMHE) was created to respond to this public health crisis. It follows a similar social-ecological approach as suggested by the NIH-convened expert panel (17) (Figure 1). SCMHE is one of 10 research centers to receive funding from the NIH-sponsored Implementing a Maternal Health and Pregnancy Outcomes Vision for Everyone (IMPROVE) initiative that supports research on how to reduce preventable causes of maternal morbidity and mortality while improving health equity. This 7-year award will fund three R01-equivalent research projects, a training core, and a community core that comprise the SCMHE.

### Leadership structure and partnering organizations

Three organizations equitably co-lead SCMHE: Reproductive Health (RH) Impact, an advocacy community-based organization; Tulane University, an academic research institute; and Ochsner Health, a large regional non-profit health system. SCMHE Principal Investigators are affiliated with each of these three entities. This triumvirate leadership model reflects each organization's complementary strengths and unique resources to cohesively address affected communities.

RH Impact, founded and run by Black women, creates solutions to optimize Black maternal and infant health through training, research, policy advocacy, and community-centered collaboration. RH Impact became fiscally sponsored by The Praxis Project in 2023. The Praxis Project is a national non-profit organization that works in partnership with national, regional, state, and local partners to achieve health equity and justice for all communities. The Praxis mission is to build healthy communities by transforming the power relationships and structures that affect Black lives and communities. RH Impact partners with hospitals and health systems in a quality improvement and technical assistance role. To date, RH Impact has trained over 2,000 maternal and child health practitioners and stakeholders to curb the impact of racism, classism, and gender oppression, and to optimize Black maternal and infant health outcomes. RH Impact will address the gaps in communication and collaboration between health systems and Black communities who are most affected by perinatal health inequities in the Gulf South.

Tulane University, a renowned research institution, will ensure technical quality and rigorous data collection and study design of the research projects. Additionally, building on Tulane's commitment to student service learning and workforce training, Tulane, in conjunction with RH Impact, will establish a synergistic, rigorous, and multilevel training program in women's health and health disparities for graduate students and junior faculty.

Ochsner Health is a non-profit healthcare system that operates 46 hospitals and more than 370 health and urgent care centers across Louisiana, Mississippi, and the Gulf South. Ochsner Health brings strength in digital medicine and technological innovation and ensures clinical relevance, as well as feasibility for future implementation and access to populations in the Gulf South for recruitment. In 2022, approximately 30% of the births in Louisiana occurred in an Ochsner facility.

These three organizations share equitable and meaningful partnerships throughout research and training activities. Each organization jointly developed all project goals and structures, has a leadership role in each component of the Center, and receives significant budget distributions.

The Center further incorporates the goals and roles of many long-term community partnerships through its additional partnerships with the University of Mississippi Medical Center, Dillard University, and community groups like Crescent City Family Services, Birthmark Doulas, and the New Orleans Maternal and Child Health Coalition. Before the bulk of the research commences at the beginning of Year 3, significant time will be spent convening the community, academic, and healthcare partners to establish agreements, standards, and values for equitable partnerships throughout the project and the Center's duration. There will be regular, bidirectional training, interpretation, and dissemination among all partners.

### Integrated approach

**Figure 2:**
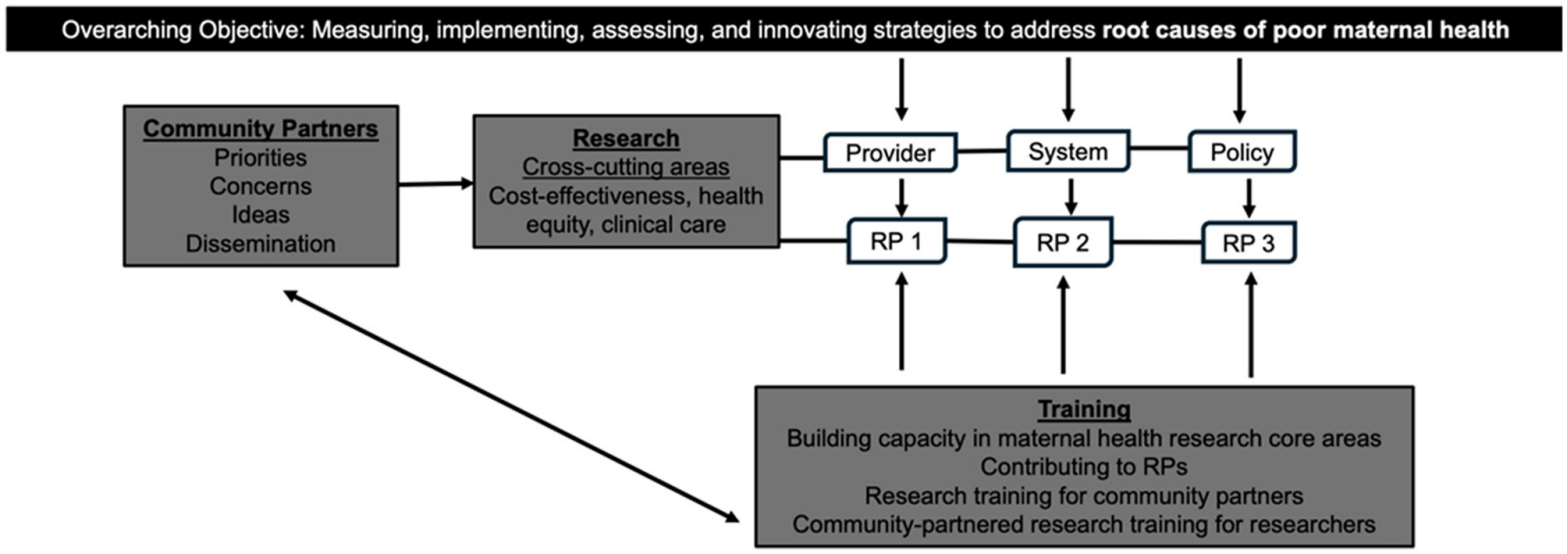
SCMHE organization.

The three integrated research projects (RP1, RP2, RP3) and Research Core (RC) provide infrastructure to support innovative and collaborative work among the investigators and internal and external partners that will further benefit from the methodological, technological, and theoretical advancements catalyzed by the Center (Figure 2). There is a 2 year pilot phase followed by a 5 year implementation phase. Throughout the 7 year program, cross-cutting specialties like cost-effectiveness analysis, equity analysis, and clinical care ensure that the multilevel projects take advantage of joint efficiencies and are evaluated as a unified whole.

Our collaborative structure incorporates community members in all facets of the Center, enabling a fundamental shift toward centering the lived experiences of historically and currently marginalized populations and uplifts community assets known to effectively improve maternal outcomes. Our strong community-partnered projects will result in sustainability and capacity building of health promotion organizations, with whom we have existing robust relationships, positioning them to monitor our efforts and build upon the programming added through the Center. Innovative programs will be continually evaluated and updated through the input of our community partners. The Training Core will build a strong cadre of minority researchers focused on maternal health via a rigorous, interdisciplinary, and community-centered strategy that aims to equip emerging investigators from populations underrepresented in the health sciences with competencies that address the biological, behavioral, environmental, sociocultural, and structural factors that affect maternal morbidity and mortality. The overall program evaluation and practice-based approach ensures scalability and direct translation of findings through our Community Partners Core.

### Theoretical framework

The Center will apply the six principles of community-led research for reproductive justice (19) to assess systemic inequities that contribute to maternal morbidity and mortality through intentional collaboration and power shifting (Table 1). Specifically, in Year 1, community members who directly serve marginalized community members most affected by maternal morbidity and mortality will be recruited into a community advisory board or community action committee. Community partners will receive a regular stipend for their role in helping to facilitate equitable and collaborative partnerships throughout the Center. Community members will be recruited based on their experiential knowledge and connections to their communities. Strengths within the community will be uplifted. Community members will ensure the tenets of reproductive justice are upheld throughout all aspects of the Center. Community members will also be essential to disseminating results in ways that are meaningful to their communities.

**Table 1:** Organization of SCHME by objectives and aims.

	**Objectives**	**Aims**	**Principle of community-led research for reproductive justice**
**Overall**	Improve maternal health through testing community-prioritized strategies that improve maternal health and strengthen community-based maternal health research capacity in Louisiana and Mississippi.	Establish a maternal health research center embedded within ongoing community partnerships and the existing maternal health research infrastructure to build and strengthen the network for improved maternal health in the Gulf South and nationally.	1. Center the marginalized community members most affected by maternal health inequities as leaders of research. 2. Facilitate equitable, collaborative partnership through all phases of maternal health research. 3. Honor multiple ways of knowing (experiential, cultural, empirical) for knowledge justice and cross-directional learning across the team. 4. Build on strengths (not deficits) within the community. 5. Implement the tenets of reproductive justice including structural-level analysis and the human rights framework. 6. Prioritize disseminating useful findings to community members first then to other audiences.
**Research Core**	Assess social, structural, and policy interventions that combine to create a multilevel approach to advancing maternal health equity.	Test multilevel strategies for improving maternal health at the individual, interpersonal, community, and policy levels via three research projects.	
**Research Project 1**	Demonstrate a multifaceted, evidence-based intervention will reduce hospital disparities in maternal outcomes.	Determine the effect of a multifaceted respectful maternity care intervention on hospital maternal outcome and patient satisfaction disparities.	
**Research Project 2**	Examine the potential of enhanced remote care for pregnancy-induced hypertension to improve maternal health.	Test implementation, process, and health outcomes of the enhanced remote care package.	
**Research Project 3**	Elucidate and evaluate effective implementation strategies that aim to improve maternal postpartum health in a vulnerable population with high risk of cardiovascular disease.	Examine the overall effectiveness of Medicaid postpartum extension (Louisiana, Mississippi) and postpartum depression screening mandate (Louisiana) on the rates of biomedical markers of maternal morbidity and healthcare utilization.	
**Training core**	Build maternal health research capacity via innovative educational programming, emphasizing bridging diverse scholars to research positions.	Develop innovative and transdisciplinary educational and training opportunities for early career and junior scholars aimed at developing a research workforce that can reduce maternal morbidity and mortality locally, regionally, and nationally.	
**Community partners core**	Incorporate community perspectives into all aspects of the research and training components and translate research findings to the community for their advocacy and other efforts.	Integrate community priorities, vision, and expertise in all areas of the project, and to return results and benefits of research and training directly to impacted communities.	

### Objectives and aims

The Center's overarching goal is to address root causes (social, structural, policy) of maternal morbidity and mortality, with a focus on Black women in the Gulf South. The overall objective of SCMHE is to improve maternal health through testing the implementation of community-prioritized strategies that improve maternal health and strengthen community-based maternal health research capacity in Louisiana and Mississippi. To accomplish this objective, SCMHE will use an intersectional lens as it applies the principles of reproductive justice to its aims. SCMHE aims to establish a maternal health research center embedded within our ongoing community partnerships and the existing maternal health research infrastructure to build and strengthen the network for improved maternal health in the Gulf South and, in collaboration with the other maternal health Centers of Excellence, across the country. The SCHME is organized into a Research Core, Training Core, and Community Core each with its own objectives and aims (Table 1).

## Methods and analysis

### Study design

The SCMHE is a community-centered research center to improve maternal health and strengthen community-based maternal health research capacity in Louisiana and Mississippi. The SCMHE applies the multilevel life course conceptual framework (Figure 1) to prevent maternal morbidity and mortality with interventions at individual, interpersonal, community, and societal levels. As detailed in the following sections, components of the SCHME simultaneously address factors contributing to maternal morbidity and mortality at multiple levels.

#### Research project 1 (a multifaceted intervention to address bias in maternal outcomes)

Mistrust rooted in a history of discrimination, experimentation, and stereotyping contributes to the present-day racial inequities in maternal morbidity and mortality (20, 21). Patient surveys and personal stories leave no doubt that implicit bias and racism in the healthcare system continue to contribute significantly to entrenched and worsening maternal health inequities (22, 23). In response to growing reports of unfair treatment in maternity care settings experienced by women of color (24), there has been nationwide momentum and adoption of interventions to address implicit bias. However, most have been targeted at an individual level with one-time training sessions. Given the depth and pervasiveness of racial bias in US culture, it is unlikely that a single, simple intervention will be enough to counter centuries of anti-Black history.

Moreover, despite their outsized risk and knowledge derived from lived experience, Black people are often excluded from the very research designed to improve their health and health outcomes (25). Effective maternity care interventions require a foundation of research evidence guided and co-led by community organizations that fill the gap in collaboration between health systems and Black communities, as is the case in Research Project 1 (RP1). Establishing the community as their own experts, community organizations will work in partnership with hospitals to develop a warm hand-off strategy for patients from the hospital to their community. This warm hand-off will embed access to social services and resources in hospital protocols, electronic medical record systems, and networks. The warm hand-off to community will establish a network of community-based organizations and partners who will provide a range of services and meet the needs of patients.

A staff patient navigator (e.g., nurse, social worker) will be established at each hospital site to coordinate with community for the warm hand-off from hospital to community. The patient navigators will work in partnership with the project team and community action committee members for training and ongoing support. The details of the role, requirements, and practical implementation duties of the patient navigator will be developed in partnership with community partners, Ochsner, and the project team. This will ensure that the role of the patient navigator is tailored to the specific needs of each community. The staff patient navigator will ideally be someone with lived experience, but lived experience will not be a requirement to avoid overburdening those who represent groups of marginalization or those with lived experience. The number of patient navigators and who fills that role will ultimately be determined by the hospital's administration. The warm hand-off to the community has been demonstrated to be successful across many variables (26).

Therefore, Research Project 1 (RP 1) hypothesizes that an interactive, multifaceted respectful maternity care intervention will reduce provider biases and improve patient satisfaction and health outcomes. The study design is a cluster randomized controlled trial that will compare the intervention—including community-led implicit bias training, technical assistance for the embedding of equity in quality improvement initiatives and building community partnerships—to a remote asynchronous training alone. Components of the intervention target individual, interpersonal, institutional, and community levels.

#### Research project 2 (connected MOM+: implementing a remote blood pressure monitoring program in areas with low maternity care availability)

There are entrenched racial inequities in receiving treatment for hypertension and hemorrhage—leading causes of pregnancy-related mortality (15). In Louisiana, those living in parishes (counties) that lack access to maternal care [i.e., have no birthing facilities or maternity care providers (27)] experience a 91% increase in risk of death during pregnancy and up to 1 year postpartum, independent of how urban or rural, impoverished or underinsured the parish, or the woman's age, race/ethnicity, or socioeconomic position (16). Black women are already at high risk independent of their residential status (28–31).

When pregnant patients live far from care sites, remote care reduces the need for long transportation, limits unnecessary care, and identifies problems earlier. This project will test an enhanced version of the existing Ochsner's Connected MOM program (at-home blood pressure). Connected MOM was developed through Digital Medicine within the Ochsner system and has gone through multiple iterations and technological challenges have been overcome. Connected MOM has been successfully implemented in southern Louisiana and has been associated with a reduction in preterm births. However, Black and rural women were less likely to participate in the program than White and urban women. Thus, Connected MOM was chosen as a program to improve and expand because baseline data on effectiveness and uptake exists and therefore allows for a more detailed study on implementation with the goal of improving equity.

Research Project 2 (RP2) hypothesizes that building upon the Connected MOM program will more effectively address barriers to care and reduce racial and geographic inequities in maternal health. In the formative Phase 1 (years 1–2), we will collaborate with community groups to develop the enhanced remote care package, adapting the Connected MOM program to the local context. This will include addressing logistical barriers (i.e., internet access) and adding additional community-based support to ensure that the intervention meets the needs of Black pregnant and postpartum women living in rural areas. The RP2 team will regularly meet with clinicians and community members from the areas of focus, and conduct formal in-depth interviews and focus groups, to determine the experiences of previous users and non-users and barriers to uptake. The intervention protocol will be based on these interviews and meetings to identify strategies to overcome barriers and gain community support. The protocol will be reviewed by the community members from the community partners core, clinicians in the implementation areas, and other staff, community, and potential patients, and revised after receiving their input. Phase 2 will implement and test the enhanced intervention, using a stepped-wedge design to assess care access, health, implementation, and patient-reported outcomes. Together, components of the intervention target individual, institutional, and community levels.

The formative and evaluative interviews also include questions about what other maternal health issues are of concern to patients, clinicians, and community members, and whether blood pressure is seen as a priority. This additional information is being gathered to return to the health care system and to Ochsner Digital Medicine specifically for their consideration of possible extensions or additions in the future.

#### Research project 3 (impact of Medicaid postpartum coverage extension and mandated postpartum depression screening on care for gestational diabetes and pregnancy-induced hypertension)

Hypertension during pregnancy (HDP) and gestational diabetes mellitus (GDM) are leading causes of maternal morbidity in the country and in the region (32–39). They are also conditions with implications for later-life health, so postpartum follow-up would be particularly important to ensure that people are connected to health care to prevent later complications. National recommendations currently recommend that all patients with hypertensive disease in pregnancy receive follow up bp evaluation within 7 days postpartum and then again at 4–6 weeks postpartum (40). Similarly, for all patients with GDM, a glucose tolerance test for evaluation of the presence of previously undiagnosed GDM is recommended at 6 weeks postpartum or the cessation of breastfeeding (40). Up to 50% of patients with GDM will be diagnosed with type 2 diabetes mellitus within 5 years in the absence of lifestyle modification (41–43).

Access to healthcare is also dependent on healthcare coverage. While Medicaid covers nearly half of deliveries in the US, the standard structure of state Medicaid policies creates a coverage gap after 60 days postpartum, where recipients cycle out of Medicaid coverage based on pregnancy. However, the American Rescue Plan Act of 2021 gave states the option to extend postpartum coverage for up to 12 months and continue to receive federal matching funds (44, 45). Louisiana was approved for Medicaid postpartum extension (MPE) in 2022 and Mississippi was approved in 2023. Louisiana also mandates screening for postpartum depression (PPD) and other mental health conditions, effective August 1, 2022, whereas Mississippi does not. Mental health is a leading cause of postpartum morbidity and mortality (10).

Research Project 3 (RP3) targets the policy level. RP3 hypothesizes that the MPE (Louisiana, Mississippi) and PPD screening mandate (Louisiana) are associated with improved maternal morbidity outcomes and healthcare utilization. The study design is a rigorous quasi-experimental approach to evaluating these policy strategies aimed at improving maternal postpartum health by comparing trends in Louisiana and Mississippi where the policies have been implemented, to trends in Arkansas where no such policies are in effect. Furthermore, longitudinal interviewing will be used to explore the lived experiences, perspectives, and opinions of Medicaid beneficiaries on the healthcare they receive during pregnancy through 12 months postpartum. Interviewing and concept mapping will be used to explore complex contextual factors impacting policy adoption (non-adoption) and implementation and the state, healthcare system, and provider levels.

#### Training core

The Training Core seeks to improve diversity among health researchers and build maternal health research capacity via innovative educational programming at multiple levels. The Training Core will support the development of a well-prepared group of researchers focused on health equity who are drawn from populations both historically underrepresented in the biomedical research workforce and deeply impacted by pregnancy-associated morbidity and mortality to support innovative solutions to address maternal morbidity and mortality.

Three to six early career researchers—defined as current doctoral students, post-doctoral graduates, and junior faculty—will be recruited from the partner institutions to participate in a multimodal training program that incorporates experiential training, professional and peer mentorship, and career development. After being added to the team of one of the center's research projects, each trainee will be matched to a primary mentor within their corresponding research project to guide their research experience, a community mentor from the Community Partners Core's Community Advisory Board to provide guidance on centering community in their research, and an executive mentor from the senior leadership of the center's executive team to provide high-level career guidance. The mentor team and their designated trainee will work together to develop a full Career Development Plan with benchmarks oriented to the trainee's transition toward an independent research career, including developing research and presentation skills, community engagement, and classroom education. In addition to their experiential learning, the trainees will also be required to attend bimonthly career development workshops such as Career Planning for Tenure and Promotion, Budget Development and Management, and Success Toward Research Independence.

The Training Core will also include a quarterly Perinatal Seminar series and an Annual Community Engagement Symposium. These events will highlight innovative perinatal health research and will include the work of the trainees. Trainees will be required to lead at least one seminar per year and present their progress at the Annual Community Engagement Symposium. The cross institutional team science approach of the Training Core will provide the trainees with a strong knowledge base and skills that will enable them to carry on the overall objective of the Southern Center for Maternal Health Equity—improving maternal health through community-prioritized strategies.

#### Community partners core

The Community Partners (CP) Core will incorporate community partners who are embedded within the priority population as invaluable collaborators with the research and healthcare cores of the Center. Community partners will include, among others, healthcare providers (e.g., doulas and midwives), those who provide social services to families, and maternal health advocates.

To elevate and incorporate Black voices throughout the Center, the CP Core will establish equitable partnerships between representatives from Black-led community-based organizations (CBOs), researchers, and healthcare providers. These equitable partnership agreements will counteract the paternalistic framework of traditional research centers that have historically excluded those who represent the targeted community. Community partners will choose an RP to work closely with and lend their expertise. Each RP will have a monthly community-focused meeting with their designated community partners (3–4 community partners per RP). For example, when strategizing about the warm hand-off of patients into their communities in RP1, community partners will work alongside RP1 researchers to connect with local CBOs and determine the best practices for the warm hand-off. Furthermore, by working with CBOs primarily led by and serving Black women, we will establish trust with and have greater access to the target population and help build these organizations' capacity and sustainability via technical assistance (e.g., training on grant writing, data collection, and data management) (46).

The CP Core will also create a research training laboratory where community partners and researchers learn in partnership and synergize their knowledge and experience, assets (e.g., community resources, access to academic institutions), and networks to achieve the overall objective. Lastly, the CP Core will help facilitate the translation and dissemination of efficacious prevention, treatment, and policy focused on reducing racial inequities in maternal health and the root causes of these disparities back to the community and a wide variety of stakeholders.

## Discussion

### Anticipated results

To advance the field of maternal health using participatory, community-centered, and radically equity-focused approaches previously underutilized and under-evaluated, the Center will assess the implementation of existing evidence-based strategies and build the evidence base for novel approaches to translation and scalability. Clinical and most implementation outcomes will be collected through administrative data, including data on maternal and infant outcomes and uptake and enrollment in the interventions. Policy-related analyses will be conducted with Medicaid claims data and electronic health record data from the participating states. Common data elements proposed by the IMPROVE initiative will be collected to the extent possible.

#### Individual, interpersonal, institutional, and community levels

Upon completion of RP1, we expect to demonstrate the efficacy of combined implicit bias training and a community warm hand-off intervention in reducing adverse maternal health outcomes, provider biases, and patient satisfaction disparities. In doing so, we anticipate illuminating how structural racism promotes disparities in treatment and care, challenges with navigating hospital hierarchies that may be detrimental to care transition, and the processes by which discrimination or bias result in harm or mistreatment. We will also explore responses to the interventions and how they shaped the participants' lived experiences and perspectives. These results are expected to have an important positive impact by providing strong evidence-based proof of principle that, by bringing together existing, theoretically based approaches for addressing biased treatment and care, maternal outcome disparities are reduced.

At the end of RP2, we expect to have a strong paradigm for implementing an enhanced Connected MOM program in locations with no or low access to maternity care across the Gulf South and beyond to reduce maternal morbidity and mortality. This will have an important positive impact by improving the health of and empowering a high-risk, underserved population to manage and advocate for their health at the individual level. Specifically, we expect to find that locations with Connected MOM+ have a greater proportion of patients attending the recommended number of prenatal visits, better medical outcomes, and higher patient activation and satisfaction. Our mixed-methods design with pre-implementation measures during intervention development and post-implementation outcomes will elucidate the complex contextual factors that contribute to the successful uptake of remote monitoring interventions in this region. This will have an important positive impact by uplifting the experience of communities who have been marginalized and are underrepresented in research as well as providing an intervention intended to improve health and care.

#### Community level

The proposed transdisciplinary educational and training program for early career and junior scholars that comprise the Training Core will strengthen and diversify a research workforce that is sustainably positioned to improve maternal health and reduce maternal morbidity and mortality through system-level changes. Trainees will develop and investigate their own research focus with support from mentors. By the end of their training, trainees will advance to the next step in their career ladder (e.g., from postdoc to faculty or from fellow to attending). In the long term, trainees will become community-responsive and independent maternal health researchers at academic, government, or other research institutions.

We expect that CP Core's involvement in the Center's research projects will lead to better-informed research programming and provide an understanding of community-centered care and more distal factors that impact maternal morbidity and mortality inequities. Through our research training laboratory, we expect to build the capacity of our partners regionally, resulting in increased community-led and -driven research that will improve sustainability and further reduce inequities in maternal morbidity and mortality in the Gulf South. Through dissemination and translation efforts, we expect that knowledge of innovative and efficacious programs and policies will grow, leading to long-term policy and practice changes. We also expect that prioritizing capacity-building efforts with our community partners will generate sustainable and system-level changes at local levels throughout the Gulf South, improving our collective efforts to change the course of these outcomes in our region.

#### Policy level

At the policy level, RP3 will demonstrate the overall effectiveness of MPE and PPD screening mandate on the rates of screening and diagnosis of type 2 diabetes mellitus, hypertension, and PPD, type 2 diabetes mellitus and hypertension controls, PPD care, disparities, and health care utilization, as compared to non-MPE and non-PPD screening mandate counterparts. We will also demonstrate the overall cost-effectiveness and distributional cost-effectiveness of MPE and PPD screening mandates in combination and separately. Using a hybrid effectiveness-implementation design, we will elucidate complex contextual factors that influence adopting and implementing MPE and PPD screening mandates, and subsequent impacts on healthcare delivery, healthcare utilization, and maternal health equity within and between Louisiana, Mississippi, and Arkansas.

Collectively, SCMHE will generate urgently needed data on practical, robust, and sustainable population-targeted strategies as well as highly proficient, culturally adept minority researchers to improve maternal health outcomes, reduce maternal health risks, and eliminate maternal health disparities in the US.

## Ethics and dissemination

### Ethics

All research projects will be approved by the Institutional Review Boards of the participating institutions. More broadly, we will consult with the community throughout the project to maximize benefits to the community and minimize issues that often arise when research is conducted by outside scientists. Rather than take the traditional paternalistic approach to academic and healthcare research, SCMHE is guided by the principles of community-led research for reproductive justice (19). This approach is unique in its commitment to addressing the crisis in the Black population and local communities alongside its community partners and upholding the expertise of its community partners to the same level as the experts within RH Impact, Tulane, Ochsner, and UMMC.

### Dissemination

By leveraging our team's *existing* network with local, regional, and national partners while continuing to build *new*, unique interdisciplinary partnerships, we will build upon our distinctive *interdisciplinary strengths*—community-rooted participatory research, individual-, peer-, and community-based interventions, social work, prevention science, community health sciences, social epidemiology, statistics—and community connections to bring our outreach, and technical assistance efforts to diverse audiences. Also, our efforts will integrate findings from cutting-edge, rigorously evaluated research strategies designed to inform maternal morbidity, and mortality prevention efforts with formative research to implement novel and cross-cutting outreach, dissemination, and translation efforts.

A distinguishing feature of our dissemination efforts is that they will be coordinated by the CP Core team in partnership with the Center Core Directors and advisory boards. An overarching aim for the CP Core is to translate the evidence base for strategies that reduce disparities in maternal morbidity and mortality into widespread outreach and dissemination efforts targeting community groups, organizations that serve Black mothers and birthing persons, hospital systems, and policymakers responsible for facilitating collaborations with outside entities across the Gulf South region. The CP Core team, including its community partners, along with input from other Center Cores, will determine the ideal types of products to develop for translation initiatives. Translational products will be culturally, linguistically, and educationally appropriate and targeted to various audiences and stakeholders. We will also provide supporting structures to community partners through translational research assistance and resources, such as how to improve cell phone coverage in rural areas.

## Conclusion

Reversing the ongoing maternal health crisis in this country is not possible without centering the Black experience. Nowhere are racial inequities and poor maternal health in starker relief than in the Gulf South, where states consistently rank last in the country on indicators of health and wellbeing. Through our unique partnership model and structure, we anticipate that the SCMHE will develop innovative approaches that will not only improve pregnancy and birth experiences but will also save lives and prevent maternal morbidity and mortality in populations and communities at among the highest risk nationally.
